# Assessment of Length and Readability of Informed Consent Documents for COVID-19 Vaccine Trials

**DOI:** 10.1001/jamanetworkopen.2021.10843

**Published:** 2021-04-28

**Authors:** Ezekiel J. Emanuel, Connor W. Boyle

**Affiliations:** 1 Department of Medical Ethics and Health Policy, Perelman School of Medicine, University of Pennsylvania, Philadelphia

## Abstract

**Importance:**

Informed consent is a fundamental element of research ethics. The COVID-19 vaccine trials are high profile trials that have enrolled more than 100 000 participants. Consent documents must be succinct and understandable to ensure informed voluntary participation.

**Objective:**

To assess how well informed consent documents of the COVID-19 vaccine trials achieve the ideal of being succinct and understandable, and to create a shorter, more readable document.

**Design, Setting, and Participants:**

This quality improvement study collected and analyzed the informed consent documents used in 4 COVID-19 vaccine phase III randomized clinical trials to quantitatively assess readability and length and, based on this analysis, created a measurably more accessible informed consent document. Analysis was conducted from October 2020 to January 2021.

**Main Outcomes and Measures:**

The main outcomes were number of words (measured as word count), time-to-read (measured at reading speeds of 175-300 words per minute), language complexity (measured using Flesch-Kincaid Grade Level assessment), and readability (measured using Flesch Reading Ease Score). Secondary outcomes included clarity of how the placebo group could access the vaccine if it is proven safe and effective. The study also examined the length and readability of an improved consent document.

**Results:**

The 4 informed consent documents were a mean (range) of 8333 (7821 to 9340) words long, with a mean (range) 35 (32.6 to 38.9) minutes to read at 240 words per minute. All documents exceeded grade 9 language complexity and scored lower than 60 in the formal reading ease metric, which constitutes difficult. Only 1 document specified that participants in the placebo group might receive vaccine. It was possible to write a document in fewer than 3000 words with a grade 7 to 8 reading level and a formal readability score that was not difficult.

**Conclusions and Relevance:**

These findings suggest that existing COVID-19 vaccine informed consent documents were too long, difficult to read, and exceeded grade 9 in language complexity. It was possible to create a shorter, more readable informed consent document for these trials.

## Key Points

**Question:**

What are the accessibility and understandability for average audiences of current informed consent documents for the COVID-19 vaccine trials?

**Findings:**

This quality improvement study of 4 informed consent documents from 4 major COVID-19 vaccine trials found all to be overly long and complex, exceeding a grade 9 language complexity and requiring a mean of 35 minutes to read the entire document. It was possible to reduce the length of the documents by more than 50% and use more broadly understandable language with a grade 7 or 8 reading level.

**Meaning:**

These findings suggest that informed consent documents may fail to succinctly explain the studies to participants of all reading levels and that it is possible to improve these documents to increase participant accessibility.

## Teaser

This quality improvement study assesses the length, readability, and complexity of informed consent documents for the COVID-19 vaccine phase III randomized clinical trials.

## Introduction

Informed consent is a fundamental protection for research participants. Informed consent documents are one, albeit critical, element in a process that is meant to ensure participant understanding and voluntary participation. Federal regulations on informed consent emphasize that documents should be brief, readable, and prioritize participants’ understanding.^1^ Yet, over time, these documents have become longer and more complex.^1,2^

The COVID-19 vaccine phase III randomized clinical trials have been the most visible clinical trials in more than 30 years and collectively enrolled more than 100 000 Americans. These trials are occurring after the revision of the federal regulations that emphasize shorter, readable documents.^3^ The goal of this quality improvement study was to examine how well the informed consent documents achieve the ideal of being succinct and understandable.

## Methods

This quality improvement study was exempt from institutional review board review because it involved no human research participants. In this quality improvement study, we systematically evaluated the informed consent documents from the AstraZeneca, Johnson & Johnson, Moderna, and Pfizer COVID-19 phase III vaccine randomized clinical trials based on 4 criteria. First, for length, we counted words and calculated the approximate time-to-read based on a typical 240 words per minute (wpm) reading speed (range, 175-300 wpm).^4^ Second, for language complexity, we used a Flesch-Kincaid Grade Level assessment.^5^ Third, for readability, we evaluated the Flesch Reading Ease Score, a 1 to 100 ranking, with scores less than 60 being considered by the Department of Health and Human Services (HHS) as difficult.^6^ Fourth, we assessed how the documents addressed access for placebo groups to the vaccine if it is proven safe and effective.

We wrote a shorter, more readable consent document. This document covers the same substantive material as the original informed consent forms.

All reading analysis was conducted using Readable.^7^ Analysis was conducted from October 2020 to January 2021.

## Results

Among the 4 informed consent documents examined, the mean (range) page count was 21.8 (17-25) pages, and the mean (range) word count was 8333 (7821-9340) words (Table 1). At 240 wpm, a participant would need a mean (range) of 34.7 (32.6 to 35.9) minutes to read an informed consent document, not accounting for rereading. Adults with slower reading ability (175 wpm) would require a mean (range) of 47.6 (44.7-53.4) minutes, if they were able to read without stopping.

**Table 1.** Characteristics of the Phase III COVID-19 Vaccine Randomized Clinical Trial Informed Consent Documents^a^

Metric	Pfizer	Johnson & Johnson	Moderna	AstraZeneca	Mean	Proposed alternative
Length, pages, No.	25	25	20	17	21.8	10
Document reading time, min						
175 wpm (lower bound)	44.7	47.7	53.4	44.7	47.6	16.9
240 wpm (mean)	32.6	34.8	38.9	32.6	34.7	12.3
300 wpm (upper bound)	26.1	27.8	31.1	26.1	27.8	9.9
Word count, No.						
Whole document	7828	8341	9340	7821	8333	2960
Risk section	884	989	1445	977	1074	200
Privacy section	2478	1955	1280	1750	1866	778
Reading grade level						
Whole document	9.8	8.8	9.6	11.3	9.9	7.6
Risks section	9.5	8.5	9.4	11.2	9.7	6.1
Privacy section	11.7	10.7	11.5	13.1	11.8	9.6
Reading ease^b^						
Whole document	52.2	56.8	51.1	49.6	52.4	61.8
Risks section	58.8	56.8	54.8	46.9	54.3	71.2
Privacy section	39.8	48.7	40.3	41.0	42.5	53.8

Abbreviation: wpm, words per minute.

^a^ Range, 0 to 100, with 100 indicating easiest to read and scores less than 60 considered difficult by the Department of Health and Human Services.

The language complexity in all the documents exceeded a grade 9 reading level, which is higher than the recommended grade 6 reading level.^6^ Additionally, all the documents had scores of less than 60 in the reading ease metric, with a mean (range) score of 52.4 (49.6-56.8), categorizing them as difficult (Table 1).^6^ Finally, only 1 document indicated that participants in the placebo group might receive vaccine. Even then, the reference was oblique, and failed to specify the timeline or other details.

Table 2 provides 3 examples of lengthy, complex descriptions that could be simplified without compromising—and maybe improving—participant comprehension. We formulated a substitute informed consent document covering the same topics that had fewer than 3000 words, with a reading time of 12.3 minutes, a reading level between grades 7 and 8, and a readability score of 61.8, which is higher than the recommended HHS threshold (Table 1; eAppendix in the Supplement).

**Table 2.** Examples of Informed Consent Language and Potential Simplifications

Document section	Original				Potential simplification			
	Text	Length, words, No.	Grade level	Readability score^a^	Text	Length, words, No.	Grade level	Readability score^a^
Purpose	Coronaviruses are a large family of viruses that cause illness ranging from the common cold to more severe disease, such as Middle eastern respiratory syndrome and SARS-CoV. Coronaviruses are zoonotic, meaning they are transmitted between animals and people. An outbreak of COVID-19 caused by the 2019 novel coronavirus SAFr-CoV-2 began in Wuhan, Hubei Province China in December 2019 and has spread throughout China and to over 200 other countries and territories, including the United States. There is currently no vaccine that has been shown to be effective against SARS-CoV-2. Therefore there is an urgent public health need for rapid development of novel interventions to prevent the spread of this disease. This study is testing mRNA-1273 study vaccine at a dose of 100 μg. The main purpose of this study is to understand if MrNA-1273 can prevent COVID-19 and to understand the safety of the mRNA study vaccine	146	12.4	43.1	This study will test a vaccine against the virus that causes COVID-19.	12	6.8	67.8
Risk	As in all research studies, the COVID-19 vaccines may involve risks that might be expected based on results from studies of similar vaccines, as well as risks that are currently unknown	31	14.4	47.1	This vaccine may cause currently unknown risks.	7	5.7	66.8
Pregnancy protections	The study doctor will discuss with you the methods of birth control that you should use while you are in this research study and will help you select the method(s) that is appropriate for you. The study doctor will also check that you understand how to use the birth control method and may review this with you at each of your research study visits	65	13.1	59.3	The study doctor will help you choose and implement an effective method(s) of birth control.	16	8.4	61.9

^a^ Range, 0 to 100, with 100 indicating easiest to read and scores less than 60 considered difficult by the Department of Health and Human Services.

## Discussion

This quality improvement study found that informed consent documents for the phase III randomized clinical trials of COVID-19 vaccines did not adhere to the widespread view about how informed consent should occur and what constitutes a good informed consent document. All the documents were long, written at a high school reading level, and would be deemed difficult by HHS.^6^ Despite their length, most documents did not inform participants in the placebo group what would happen were the vaccines proven safe and effective.

Why are these vaccine trial consent documents so long and difficult to read? While it is impossible to say definitively, we posit 3 contributing factors. First, institutional review board members may not insist on shorter, more readable documents and/or may require additional material that they think, without any supporting data, will improve information transfer or promote trust. Second, the documents may be created by copying sections from previous documents, causing the documents to grow by accretion without any effort to make them more succinct. For instance, the privacy sections in all 4 informed consent documents contained irrelevant material, and none appeared to be customized to COVID-19 vaccine studies. Third, when drafting documents, legal teams may prioritize exhaustive details or liability mitigation over participants’ comprehension.

It is possible to develop a measurably better informed consent document, one that is shorter, more readable, and uses less complex language. This requires work and effort at editing. Thus, it may be more useful for researchers to hire an editor to collaborate on creating better documents rather than leaving the creation of informed consent documents to researchers, legal teams, or others whose expertise is not careful, succinct writing.

### Limitations

This study has some limitations. Evaluating the extent to which these informed consent documents effectively communicate to ensure participants’ comprehension of each of the myriad elements of informed consent was outside the scope of this study, and this is an area that warrants additional research. It is important to concede that our rewritten form also did not achieve a grade 6 reading level. The Flesch-Kincaid Grade Level assessment depends, in part, on the syllable count of words with many syllables and the number of those words. Necessary medical terms and consent-related words without shorter substitutes can inflate the reading grade level of these documents. Thus, the appropriateness of this metric to evaluate informed consent documents merits reevaluation, perhaps with a focus on more qualitative alternatives. Such an analysis was also outside the scope of this analysis. Furthermore, we only evaluated phase III informed consent forms to allow for a more uniform comparison. While informed consent is perhaps more vital to protecting participants considering entry into earlier, riskier trial stages, the mixture of combined and stand-alone early phase COVID-19 vaccine trials compelled us to focus exclusively on documents from phase III trials.

## Conclusions

The findings of this quality improvement study suggest that participant informed consent has been compromised by lengthy, complex documents. Federal guidance has not been sufficient to create shorter, more comprehensible informed consent documents. Achieving a robust informed consent process will require a renewed commitment to documents that eliminate verbose and irrelevant material. To fulfill the goal of valid informed consent, organizations involved in clinical trials should channel the judiciousness of an editor in drafting future documents.

## Notes

Supplement. **eAppendix.** Improved, Shorter Informed Consent Document
