# Supplementary material for: Assessment of Length and Readability of Informed Consent Documents for COVID-19 Vaccine Trials
Source: JAMA Netw Open. 2021 Apr 28;4(4):e2110843. doi: 10.1001/jamanetworkopen.2021.10843 (PMC8082317; doi:10.1001/jamanetworkopen.2021.10843)
Supplement: Supplement. — eAppendix. Improved, Shorter Informed Consent Document [file jamanetwopen-e2110843-s001.pdf]

Supplemental Online Content

Emanuel EJ, Boyle CW. Assessment of length and readability of informed consent documents for COVID-19 vaccine trials. *JAMA Netw Open*. 2021;4(4):e2110843. doi:10.1001/jamanetworkopen.2021.10843

eAppendix. Improved, Shorter Informed Consent Document

This supplemental material has been provided by the authors to give readers additional information about their work.

© 2021 Emanuel EJ et al. *JAMA Network Open.*

eAppendix. Improved, Shorter Informed Consent Document

**Key Study Information and Contact Information**	The study team will address any questions or concerns you may have about the study. Here are phone numbers for the study team. **You also will be given a card with important emergency contact information.**
	Name of Study:<br>[Institution] Study Number:<br>Sponsor Study Number:<br>Name of Company Sponsoring the Study: **XXX. XX is conducting the study for XXX**<br>Name of Principal Investigator (Study Doctor):<br><br>**Study Site Contact Information:**<br>Contact Person:<br>Address:<br>Phone Number (Normal Business Hours):<br>Phone Number (Off-Hours or Emergency):<br><br>**[**Complete the following entries for the site-level ICD as appropriate.**]**<br><br>**[Institutional Review Board *or* Independent Ethics Committee] Contact Information:**<br>Contact Person:<br>Address:<br>Phone Number:<br><br>**Patient Rights Advocate:**<br>Contact Person:

© 2021 Emanuel EJ et al. *JAMA Network Open.*

	Address:<br><br>Phone Number:
**Brief Summary of this study and its purpose**	This study will test a vaccine against the virus that causes COVID-19.<br>People who participate will be randomly assigned to receive an injection either of the vaccine or of inactive salt water, a harmless placebo. People will receive 2 injections about X weeks apart.<br>The vaccine cannot give you COVID-19.<br>This study does not replace your regular medical care. If you need medical care during the study, contact your regular physician and inform the study team.<br>Taking part in this study is your choice and there is no penalty if you decide not to participate or withdraw.
Timeline and participant information for the study	You could be in this study for up to 26 months. Over XX people will take part. The study doctor will check if you meet all of the requirements to take part in this study.
Study Vaccines	If you meet the study requirements, you will be randomly assigned to receive either the study vaccine or placebo. For every 1 person who receives the study vaccine, 1 person will receive the placebo. No one– including you, your personal doctor, and the study team— can choose which you get, vaccine or placebo. You will not know whether you are receiving the study vaccine or placebo. If necessary, the study doctor can learn quickly whether you have received study vaccine or placebo.<br><br>You will be asked to wait at the study site for about 30 minutes for observation following the injection.
**Overview of Study Procedures and Assessments**	You will have to come to the study site for 6 to 7 visits. Over these 6-7 visits, you will be examined by the study doctor, receive two injections, and give blood 5 times. These steps allow the team to evaluate how your body is responding to the vaccine. If you get sick with COVID-19, more visits will be required. Each time you give blood the study team will take about 1½ tablespoons.

© 2021 Emanuel EJ et al. *JAMA Network Open.*

<table>
<tr><td>E-Diary</td><td>At Visit 1, the study team will show you how to fill in an electronic diary (or e-Diary). We will put an app on your smart phone or give you a device (a bit like a mobile phone) with the e-Diary. The data on your phone or the device is secure and will keep your data confidential. The e-Diary will ask about COVID-19 symptoms.</td></tr>
<tr><td>If You Get COVID-19 Symptoms</td><td>If you get any of the following symptoms you must contact the study doctor immediately. If you feel unwell, please contact your usual doctor and the study doctor.

• A diagnosis of COVID-19;
• Fever;
• New or increased cough;
• New or increased shortness of breath;
• Chills;
• New or increased muscle pain;
• New loss of taste or smell;
• Sore throat;
• Diarrhea;
• Vomiting.

If your study doctor suspects you have COVID-19, you will be asked to take a nasal swab that will be tested to see if you are infected with the virus. You will be asked to come back about one month after feeling unwell for another blood sample.</td></tr>
<tr><td>For people taking part in Phase 2/3, the study doctor or nurse will:</td><td>
<table>
<tr><td>Visit Number</td><td>1</td><td>2</td><td>3</td><td>4</td><td>5</td><td>6</td></tr>
<tr><td>Visit Description</td><td>Study Vaccine 1[a]</td><td>Study Vaccine 2</td><td>1-Month Visit</td><td>6-Month Visit</td><td>12-Month Visit</td><td>24-Month Visit</td></tr>
<tr><td>Ask about medical history as well as date</td><td>X</td><td></td><td></td><td></td><td></td><td></td></tr>
</table>
</td></tr>
</table>

© 2021 Emanuel EJ et al. *JAMA Network Open.*

of birth, sex, race and ethnicity						
Ask about medicines you are currently taking	X	X	X	X	X	X
Perform clinical assessment	X					
Record latest CD4 count and viral load (for HIV positive participants only)	X		X	X	X	X
Measure body temperature	X	X				
Measure height and weight	X					
Urine pregnancy test (if appropriate)	X	X				
Ask about other vaccinations you have had	X	X	X	X		
Check you meet all the study requirements	X	X				
Check contraceptives (if appropriate)	X	X	X			

© 2021 Emanuel EJ et al. *JAMA Network Open.*

Collect blood sample to test antibody levels	~20 mL		~20 mL	~20 mL	~20 mL	~20 mL
Take a nasal swab	X	X				
Get the study injection, followed by a 30 minute observation period	X	X				
Give you an e-diary or help you download one	X					
Vaccination e-diary completion for 7 days (if you are part of chosen group to report potential side effects following vaccination)	X	X				
COVID-19 illness e-diary completion	X	X	X	X	X	X
Ask how you are feeling generally	X	X	X	X	X	X

a. This visit may be conducted across 2 consecutive days

**Vaccine Risks**	The vaccine cannot cause COVID-19.<br>Reactions at the site of injection may include redness, itching, pain, and swelling.<br>Other vaccine side effects may include fever, sweats, headache, tiredness, chills, loss of appetite, muscle aches, and joint aches.

© 2021 Emanuel EJ et al. *JAMA Network Open.*

	You might have an allergic reaction which could range from mild to more severe— from a rash to swelling of the face or lips, or shortness of breath.<br>A very severe allergic shock could occur very rarely.<br>This vaccine may cause currently unknown risks.<br>If you catch COVID-19, could the vaccine make it worse?<br>We do not know whether this vaccine could make COVID-19 illness more severe.
**Placebo Risks**	The placebo injection is salt-water with no active ingredients. You can have swelling, redness, and pain at the injection site. Other side effects are less likely.
**Other Risks from Study Procedures**	Collection of blood samples and nasal swabs can cause discomfort and some pain, but cannot cause serious side effects.<br><br>**You must report all symptoms and side effects to the study team as soon as they occur. Phone numbers for the study team are listed in [Section 1] of this consent document.**
What are the possible benefits of participating in this study?	You may not directly benefit from participating in this study. It is not known whether the vaccines will reduce your chances of getting COVID-19 or how severe the disease might be.
**Pregnancy**	If you are currently pregnant, plan to become pregnant, or are breastfeeding a child, you should **not** join this study. If you are a woman who is able to have children, you will have a pregnancy test to check that you are not pregnant before you can get the study injection.<br><br>If you are male or female and able to have children and are sexually active, you must use effective birth control consistently and correctly for at least 4 weeks after you receive your second injection. Men as well as women must use effective birth control. The study doctor will help you choose and implement an effective method(s) of birth control.<br><br>**If you or your partner becomes pregnant during the research study, tell the study doctor right away.**

© 2021 Emanuel EJ et al. *JAMA Network Open.*

	**If you or your partner want to stop your required birth control during the research study, tell the study doctor <u>right away</u>.**<br><br>**Pregnancy Follow-up**<br><br>If you or your partner become pregnant up until 6 months after your last study injection, tell the study doctor right away.
**What will happen to my blood and nasal swab samples?**	Your blood and nasal swab samples will be used only for scientific research. The samples may be stored for 15 years after the study ends for future testing. No testing of your DNA will be performed. You will not be told of additional tests, and you will not receive results of any of these tests.<br>You may request that your samples be destroyed at any time. Any data already collected from those samples will still be used for the study.
**What other choices do I have if I do not join this study?**	You do not have to take part in this study. The vaccine is not yet available to the public. It will be made available if it is found to be safe and effective.
**What happens if I am injured during this study?**	If you experience an injury or illness caused by the study, your study doctor will provide or arrange for medical treatment. XXX/XX will cover the costs of this treatment. You will not receive payment for such things as lost wages, expenses other than medical care, or pain and suffering.
**What if I join this study and then change my mind?**	You are free to stop participating in this study at any time. Your decision will not affect your regular medical care or any benefits to which you are entitled. If you decide to stop participating in this study, you must notify the study doctor. The study team will explain what other procedures or discussions would occur.<br><br>The study team will tell you in a timely manner if new information is learned that could change your mind about continuing participation.<br><br>Sometimes the study doctor or XXX/XX may decide to take you out of the study even if you do not agree.
**What will I have to pay for if I take part in this study? Will I be paid or otherwise compensated?**	You will not pay for any of the study vaccines (COVID-19 vaccine or placebo), study-related procedures, or study visits.

© 2021 Emanuel EJ et al. *JAMA Network Open.*

	You will be reimbursed $XX.XX by the research site for each visit you complete. This money covers your travel and parking. You also will be paid $X.XX for each weekly illness diary you complete.<br><br>If you are in the placebo group and the vaccine is found to be safe and effective by the FDA, it will be provided to you at no cost. Initially the vaccine will be in short supply and only particular groups will be eligible to get the vaccine. The study team will give you the vaccine only when your group, such as health care workers or people over 65, are eligible in the broader society. The study team will reach out to you in the event that this happens in order to coordinate your injection.<br><br>Receiving the vaccine does not mean you are fully immune to getting COVID. Even after receiving the vaccine, you should continue to abide by public health guidelines that apply where you live.
**What will happen to keep my personal information private?**	To be part of the study, you will need to authorize the use of your personal information. But we will not publicly disclose any of it.<br><br>**A. What personal information will be collected about me during this study?**<br><br>• **Information that directly identifies you**, such as your name, address, telephone number, email address, and date of birth.<br>• **Sensitive personal information,** such as your medical history, data from this study, HIV status, race, and ethnicity.<br>• **Data from testing and analysis of biological samples and images**, such as results of blood tests and X-rays.<br>• **Data captured from electronic devices,** such as information from the eDiary.<br><br>**B. Who will use my personal information, how will they use it, and where will it be stored?**<br><br>These four types of information collected during this study will be stored at your study site. It may be sent to a secure system so that XXX/XX employees

© 2021 Emanuel EJ et al. *JAMA Network Open.*

	or representatives can review and verify study data to evaluate the study. The information at the secure system will be deleted when the study is stopped.<br><br>Your personal information might be shared with four groups as legally necessary for 1) conducting the vaccine study, 2) ensuring it is done ethically, 3) getting governmental approval of the vaccine and, 4) if it is successful, for distributing it, including:<br><br>• Government or regulatory authorities, such as the NIH and FDA.<br>• Institutional Review Board(s) (IRB) or Independent Ethics Committee(s) (IEC) that assess the ethics of the study.<br>• People or organizations who collaborate with XXX/XX;<br>• Any organization that obtains rights to sell the vaccine under study;<br><br>These groups may contact you to follow-up on your health. For legal reasons, the study site will retain your personal information up to 15 years after the end of the study<br><br>**C. What happens to my personal information that is sent outside the study site? What happens to my biological samples and images?**<br><br>Before your information is sent outside the study site, all personal identifiers, such as your name and address, will be removed. You will not be directly identified in any article or report of the study. You will not be asked to provide consent again.<br><br>Your biospecimens will not be used for commercial profit. Nor will they be subject to whole genome sequencing.<br><br>Clinically relevant research results, including ones about you in particular, will be disclosed at the end of the trial.

© 2021 Emanuel EJ et al. *JAMA Network Open.*

	**D. Can my personal information be used for other research?**<br><br>Yes. Your information may be used to advance scientific research and public health in future projects. The privacy of your information will be safeguarded such as by removing your name and other personal information and storing with security measures.<br><br>If your personal information is transferred by XXX/XX to other countries, XXX/XX, will take steps to make sure the information is kept private and the transfer is done as the law requires.<br><br>**E. What are my data protection rights? Whom may I contact about these rights or any concerns or complaints?**<br><br>You have the right to access your personal information that is held about you by the study team. *But you may not be able to review some of the data until after the study has been completed.*<br><br>If you have concerns about your data's privacy contact the study site.<br><br>**F. What happens if I stop taking part in the study?**<br><br>If you stop taking part in the study but do <u>not</u> tell the study team and withdraw your consent, your personal information will continue to be used and you may be contacted to check whether you wish to continue in the study. If the study site is unable to reach you and if the law allows, XXX/XX may use publicly available records about your health to monitor the long-term safety of the vaccine<br><br>If you decide to withdraw your consent:<br><br>• You will no longer be able to participate in the study.<br>• No new information or samples will be collected about you or from you by the study team.

© 2021 Emanuel EJ et al. *JAMA Network Open.*

	• The study team may still need to report any safety event that you may have experienced due to your participation in the study to XXX/XX.<br>• Your personal information that has already been collected up to the time of your withdrawal will be kept and used by XXX/XX to determine the safety effects of the study vaccine and to satisfy legal requirements.<br>• Your personal information will **not** be used for further scientific research.<br><br>You can request that any of your remaining biological samples be destroyed. However, we cannot guarantee the destruction of samples as they may no longer be traceable to you.
**Where can I find additional information about this study or the study results?**	A description of this clinical trial and summary of the results will be available on http://www.ClinicalTrials.gov. When available, the study results will also be available at **www.XX.com**.
**Signatures**	**Agreement to Participate and to Process Data**<br><br>1. I confirm I have read (or, a study team member has read to me) and understand this consent document for the COVID-19 vaccine study and have had the opportunity to ask questions. I have had enough time to review this consent document, to ask about the details of the study and to decide whether or not to participate.<br>I agree to take part in the study described in this document.<br><br>2. I understand and agree to the use of my personal information. I understand that my information may be used within and outside my country of residence for health care, medical research, and/or legal purposes.

© 2021 Emanuel EJ et al. *JAMA Network Open.*

	3. I understand that taking part is voluntary and that I am free to stop taking part in this study at any time. I understand I can withdraw my consent to the use of my personal information. Even if I withdraw, my personal information held at that time may be kept to comply with laws and to evaluate the vaccine study.<br>4. I agree to the study team accessing my medical history. I understand that this includes information from medical records and test results and any medical treatment I receive during the course of the study. I understand the study team may contact my doctor or any other health care providers treating me for access to such information.<br>5. I understand that XXX/XX and/or others working with or on behalf of XXX/XX, institutional review boards (IRBs) or independent ethics committees (IECs), and governmental agencies may need access to my personal information. I agree that they may have access to my personal information.<br>6. I do not give up any of my legal rights by signing this consent document.<br><br>____________________________________<br>Printed name of participant<br><br>____<br><br>Signature of participant

© 2021 Emanuel EJ et al. *JAMA Network Open.*

	Date of signature§<br>(If no legally acceptable representative is used)<br><br>§Participant must personally date their signature.<br><br>**Person Obtaining Consent:**<br><br>____________________________________<br>Printed Name of the Person Conducting the Consent Discussion<br><br>____ ________________<br>Signature of the Person Conducting the Consent Discussion †<br>Date of signature<br><br>†The investigator must sign and date the consent document during the same discussion when the participant signs the consent document. Alternatively, the investigator can designate an appropriately qualified and trained person to conduct the informed consent process.

© 2021 Emanuel EJ et al. *JAMA Network Open.*
